# Psychotic experiences in adolescents: risk factors, persistence and transition to clinical Phenotypes

**DOI:** 10.1192/j.eurpsy.2026.10322

**Published:** 2026-07-31

**Authors:** M. Di Vincenzo

**Affiliations:** Department of Biomedical and Neuromotor Sciences, Alma Mater Studiorum - University of Bologna, Bologna, Italy

## Abstract

**Abstract:**

Psychotic experiences (PEs) are subclinical psychotic symptoms observed in the general population, particularly during childhood and adolescence, where they are typically transient. However, persistent PEs associated with functional impairment confer an increased risk of transition to clinical psychosis. This presentation will examine genomic and environmental risk factors for persistent and distressing PEs in a large longitudinal adolescent cohort.

**Disclosure of Interest:**

None Declared

